# Does application of honey improve surgical outcome in pilonidal cyst excision with secondary intention healing? A prospective randomized placebo-controlled clinical trial

**DOI:** 10.1186/s13741-021-00237-w

**Published:** 2022-01-10

**Authors:** Vahid Salehi, Mohammad Javad Yavari Barhaghtalab, Saadat Mehrabi, Aida Iraji, Seyed Alimohammad Sadat, Seyed Hadi Yusefi, Jan Mohamad Malekzadeh

**Affiliations:** 1 Department of General Surgery, Shahid Beheshti Hospital, Yasuj University of Medical Sciences, Yasuj, Iran; 2 Stem Cells Technology Research Center, Shiraz University of Medical Sciences, Shiraz, Iran; 3 School of Nursing, Yasuj University of Medical Sciences, Yasuj, Iran; 4 Department of Nutrition, School of Health, Yasuj University of Medical Sciences, Yasuj, Iran

**Keywords:** Honey, Surgical outcome, Wound healing, Pilonidal cyst, Clinical trial

## Abstract

**Background:**

Pilonidal sinus disease (PSD) is a common chronic inflammatory debilitating illness caused by ingrowth of hair into the skin. Excision and healing by secondary intention is one of the acceptable managements. The post-operative wound care needs frequent and time-consuming follow-ups. Honey is considered to be a traditional remedy for wound healing. The current study aimed at finding if application of honey could improve surgical outcome in pilonidal cyst excision with secondary intention healing.

**Methods:**

This study was designed as a randomized placebo-controlled parallel assignment interventional (clinical trial) study conducted at the surgical ward of Shahid Beheshti Hospital affiliated to the Yasuj University of Medical sciences, Yasuj, Iran, and was consisted of the 48 patients who underwent surgical resection for PSD with secondary intention healing (24 patients in intervention and placebo-controlled groups). The main element of honey medicinal gel was the unheated natural honey of Dena Biosphere Reserve within the Zagros Mountains. Patients' wounds were visited by a surgeon and a nurse on the days 7, 15, 30, 45, 60, and 90 post-operation. The surgical outcomes including the time to complete wound healing, pain intensity, odor, discharge at the site of surgery, use of analgesics, the time of to return to the daily activities, and occurring of any side effects including infection, erythema, and bleeding were all recorded.

**Results:**

In intervention group, there was significantly lower wound healing time, the lower time to return to the daily activities, lower mean wound volume at the days of 30, 45, 60, and 90 of the follow-up, higher mean post-operative pain level at the days of 15, 30, 45, 60, and 90 of the follow-up, and more usage of analgesics at the days of 15, 30, 45, and 60 of the follow-up. There was no significant difference between intervention and placebo-controlled groups according to the foul smell and fluid discharge at the site of the operation. There were no side effects and complications in both groups of the study.

**Conclusions:**

Application of honey after resection surgery with secondary wound healing is associated with a better surgical outcome and could eventually decrease healing time and reduce duration of return to normal activities, but could increase post-operation pain and analgesic consumption, and no effect on foul smell and discharge.

**Trial registration:**

The project was found to be in accordance to the ethical principles and the national norms and standards for conducting research in Iran with the approval ID and date of IR.YUMS.REC.1399.088 and 2020.05.30 respectively, and is the result of a residency dissertation to get the specialty in general surgery, which has been registered with the research project number 960508 in the Vice Chancellor for Research and Technology Development of Yasuj University of Medical Sciences, Yasuj, Iran, URL: https://ethics.research.ac.ir/EthicsProposalViewEn.php?id=144742

## Background

Pilonidal sinus disease (PSD) is a common chronic inflammatory debilitating illness caused by ingrowth of hair into the skin. It is mainly a cavity with accumulated pus linked to the skin by a sinus track with granulation tissue lining (Woo et al., 2015; Salih et al., 2018). The estimated incidence of PSD is 26 to 700 per 100,000 population and male to female ratio is almost 4 to 8 (Woo et al., 2015). Obesity, long periods of sitting, increased sweating, local hirsutism, deep gluteal clefts, and poor hygiene are the risk factors for the development of the PSD (Kuckelman, 2018). The diagnosis of PSD is made clinically by the patient’s history and physical examination of the gluteal cleft. Suppurative hidradenitis, perianal abscess and fistula, infected skin furuncles, Crohn’s disease, tuberculosis, syphilis, and actinomycosis are the differential diagnoses. Midline pits and to a lesser extent hair or debris extruding from the openings are the characteristic presentations of the disease on the physical examination of the gluteal cleft. Moreover, cellulitis or a painful, fluctuant mass indicating the presence of an abscess can be seen in acute setting, and a chronic draining sinus with recurrent episodes of acute infection can be seen in chronic state (Steele et al., 2013).

PSD is mostly treated by surgery. Incision and drainage, excision and primary closure, excision and healing by secondary intention, and excision with reconstructive flap techniques are the most common surgical measures (Mahmood et al., 2020; Singh et al., 2017). Surgeons agree that for the complete treatment, all the involved skin and subcutaneous tissue should be excised and removed. However, they have not agreed whether the resulting wound should be stitched closed or left open to heal by the secondary intention. Advantages of secondary wound healing are allowing the wound to drain adequate and granulate, a lower recurrence rates and duration time of hospitalization, in contrary to more duration of healing time, needing rigorous wound care, uncomfortable dressing changes, and significant social and economic disability as the disadvantages of this procedure, resulting in a substantial morbidity (Salih et al., 2018; Kuckelman, 2018; Vermeulen et al., 2005).

Wound care after excision of PSD with healing by secondary intention leads to frequent and time-consuming post-operative follow-ups [3)]. The best dressing for post-operative wounds healing by secondary intention is unknown. There are only a few poor-quality trials evaluating the usefulness of dressings and topical agents on such wounds. A safe, simple, and effective dressing with early wound healing and cure rate, less absence from work and duration of the hospital stay, more patient satisfaction, low-cost and patient discomfort and pain, and good quality of life are the ideal criteria for post-operative care (Kuckelman, 2018; Singh et al., 2017). Retention of the moisture, absorption of the exudate, insulation of the wound bed preventing the bacterial infiltration, promotion of the normal cellular activity in an isotonic and nontoxic environment, sealing the skin in the natal cleft between the distal wound edge and the anus to prevent migration of hairs and fecal matter into the wound bed, considerable decrease in nursing time, being cost-effective, increasing patient well-being and having a faster healing times are the criteria of an advanced wound dressing. A stable and healthy wound with complete replacement with granulation tissue and re-epithelialization, being free of inflammation, infection, and necrotic tissue, having moisture balance (exudate control and wound bed hydration) and the healthy edges are the goals of the treatment (Harris & Holloway, 2012).

Special local wound care is needed for the patients with the open wounds with healing by the secondary intention, and consisted of four main categories: iodine preparations, silver, polyhexamethylene biguanide (PHMB), and the honey. The best dressing for each wound is selected according to the frequency of dressing changes needed, amount of exudate, and the patient’s self-care. A potent antimicrobial agent is the Iodine with the best penetration due to small molecular size, easy to apply, and no reported resistance; but endorses inflammation, and should be used carefully in patients with thyroid disease, and should not be used on large areas for a very long time. A good disinfectant and antiseptic available in the forms of the foam and gauze is PHMB (chlorhexidine), which keeps moisture in the wound, and is less toxic than other antiseptics. An anti-infective and anti-inflammatory agent is the silver, which is easy to use as different forms with varying silver release and moisture balance capabilities like solution, foam, hydrogel, hydrofiber, cream, or alginate dressing, but may prompt apoptosis of the keratinocytes and be inactivated by chloride and proteins (Basson & Grobler, 2008; Harris et al., 2016; Harris et al., 2012a).

Honey is a natural liquid made by bees or wasps with a sweet taste used as a natural sweetener. Honey has been described in the Torah, Bible, and the Quran. Egyptians in 2000 BC used honey in wound care for the first time, so honey is used to be a traditional, complementary and alternative medicine in wound care (Yaghoobi et al., 2013). Honey has antimicrobial effect with wound healing properties reported by several studies and is used successfully in the treatment of infantile gastroenteritis, infected surgical wounds, burns, surgical debridement, skin graft, and pressure ulcer. The healing properties of natural unheated honey are shown as “cause and effect” in Table 1 (Woo et al., 2015; Basson & Grobler, 2008; Harris et al., 2016; Harris et al., 2012a; Dorai, 2012; Yaghoobi et al., 2013; Al-Waili et al., 2011).

**Table 1:** The healing properties of natural unheated honey

Cause	Effect
Stimulation of inflammatory cytokines (TNF-α, IL-1β, and IL-6 release from Mono-Mac 6 (MM6) cells (A monocytic cell line)	Healing and tissue repair
Proliferation and activation of peripheral blood B and T lymphocytes	Healing and tissue repair
Glucose oxidase catalyzes the oxidation of glucose to gluconic acid and release hydrogen peroxide (H2O2), which causes a decrease in the honey pH and ruins the outer membrane of the bacteriae	Anti-bacterial activity and stimulates the wound healing process
Methylglyoxal	Anti-bacterial activity
Viscosity and hyperosmolarity	Anti-bacterial activity helps to absorb exudate and provide a protective barrier to prevent infection
Varying concentrations of antioxidants (flavonoids, ascorbic acid, carotenoids, catalase, peroxidase, and phenolic acids)	Anti-oxidant properties may be beneficial to wound healing
Provides a moist environment (physical properties)	Create a moist wound healing environment that does not stick to the underlying wound tissues
Acidity	Reduce protease activity, increase fibroblast activity, and increase oxygen release, assist in the bacterial-killing action of macrophages, aiding wound healing, but increase pain
Increase nitric oxide (NO) production	Affect immunity, bacterial infections, and wound healing
Inhibitory effect on prostaglandins	Affect inflammation, pain, immunity, and wound healing
Nutritional composition (glucose, fructose, sucrose, minerals, vitamins, antioxidants, amino acids, and other products)	Wide biological and therapeutic effects
Enhancing wound contracture	Healing and tissue repair
Increased granulation tissue formation	Healing and tissue repair

Little studies have been reported the local use of honey in PSD. In Table 2, six previously published reports on the use of honey in PSD are summarized (Vasei & Jahangiri, 2008; Hamdan, 2008; Grant, 2009; Thomas et al., 2011; Elhorbity et al., 2018; Hermanns & Rodrigues, 2019). In Table 3, seven previously published reports on the use of honey in other surgeries are summarized (Vasei & Jahangiri, 2008; Hamdan, 2008; Grant, 2009; Thomas et al., 2011; Elhorbity et al., 2018; Hermanns & Rodrigues, 2019).

**Table 2:** A literature review on application of honey in PSD

	Author	Country	Year	Wound Description	Number in trial	Results
1	Vasei et al. (Vasei & Jahangiri, 2008)	Iran	2008	PSD	12 patients in intervention and control groups each respectively	No significant difference between healing time patients in intervention and control groups, painful dressing, bloody oozing in half of the patients
2	Hamdan et al. (Hamdan, 2008)	UK	2008	Recurrent PSD	16 PSD patients who were all initial failures with primary treatment	Local excision and packing with honey dressing as one option in patients undergoing elective primary treatment of PSD and in those with an acute pilonidal abscess with excellent early results
3	Grant et al. (Grant, 2009)	USA	2009	Pilonidal abscess which was incised and left open for secondary healing and drainage.	3 patients	Improvement in clinical outcomes, comfortable and easy to apply dressing, managed the exudate levels, odor controlled
4	Thomas et al (Thomas et al., 2011)	UK	2011	Chronic or recurrent PSD	17 patients	15 patients with complete wound closure with significantly lower mean healing time
5	Elhorbity et al. (Elhorbity et al., 2018)	Egypt	2018	Acute infected wounds, such as wound after surgical excision of coccygeal of pilonidal sinus	50 patients in intervention and control groups each respectively	Highly effective in local management of infected wound, shortly time of healing, economic, cost effective, more patients satisfies, comfort with less pain, lesser wound scare formation, more cosmetics
6	Hermanns et al. (Hermanns & Rodrigues, 2019)	Netherlands	2019	Chronic PSD with primary closure, and then wound dehiscence	One patient as case report	Effective in preventing infection and inducing healing, reduce the prolonged or repeated use of antibiotics.

**Table 3:** A literature review on application of honey in other surgeries

	Author	Country	Year	Wound description	Number in trial	Results
1	Vardi et al. (Vardi et al., 1998)	Israel	1998	Post-surgical chronic open wound infection that failed to heal with conventional treatment	9 infants	Honey is useful in the treatment of post-surgical wounds that are infected and do not respond to conventional systemic and local antibiotic treatment
2	Al-Waili et al. (Al-Waili & Saloom, 1999)	United Arab Emirates	1999	Wound infection following caesarean Section or total abdominal hysterectomy	26 patients were treated with honey and 24 patients with local antiseptics (Ethanol and povidone-iodine)	Honey could (1) eradicate bacterial infections faster, (2) reduce period of antibiotic use and hospital stay, (3) accelerate wound healing, (4) prevent wound dehiscence and need for re-suturing, and (5) result in minimal scar formation.
3	McIntosh et al. (McIntosh & Thomson, 2006)	UK	2006	Toenail surgery with matrix phenolization	100 participants, 52 received an active manuka honey dressing and 48 received paraffin-impregnated tulle gras	Paraffin tulle gras dressings are more effective than honey dressings following partial toenail avulsion
4	Pereira et al. (Pereira et al., 2012)	Portugal	2012	A 47-year-old male patient with a loco-regional advanced right pyriform sinus tumour, with skin invasion on the anterior part of the neck, who developed with post laryngopharyngectomy wound dehiscence	Case report	Honey can be used as an alternative and experimental local therapy
5	Nikpour et al. (Nikpour et al., 2014)	Iran	2014	Cesarean section	37 cases of drug and 38 cases of placebo	Effective in healing the cesarean section incision.
6	Anyanechi et al. (Anyanechi & Saheeb, 2015)	Nigeria	2014	Benign lesions of the mandible, treated by segmental mandibular resection, developing with the surgical wound dehiscence	72 patients, 36 in control, and 36 in experimental group (dressed in honey after debridement)	Honey speeds up the healing of dehiscence wounds of resected mandible when used as dressing more than the control.
7	Goharshenasan et al. (Goharshenasan et al., 2016)	Iran	2016	Bilateral symmetric incisions in randomly selected plastic surgical patients	72 symmetrical incisions in 52 patients were randomly covered post-operatively with conventional dressing and honey dressing for 5 days	The healing process of the surgical wound and its final aesthetic result could be improved by using honey dressing.

Different types of honey vary in their effectiveness according to their anti-bacterial activity because of the plant source which honey produce from. New Zealand Manuka honey is the best worldwide known honey with the standard level of anti-bacterial activity produced from the New Zealand tea-tree or Manuka bush (Leptospermum scoparium). Medical-grade honey is available in hydrogel, hydrocolloid, and alginate preparations (Basson & Grobler, 2008; Harris et al., 2016; Harris et al., 2012a).

**Fig. 1:**
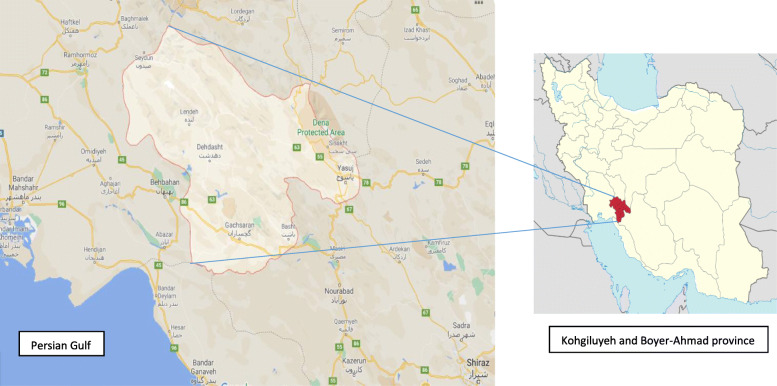
Dena Biosphere Reserve (Dena Protected Area) is located in the Central Zagros Mountains, in Kohgiluyeh and Boyer-Ahmad province in the south-west of Iran

Dena Biosphere Reserve is located in the Central Zagros Mountains, in Kohgiluyeh and Boyer-Ahmad province in the south-west of Iran (Fig. 1 (Yamaha, 2013; Map data: google@2021, n.d.)). The region has internationally noteworthy ecosystem, species, and genetic biodiversity. Dena has an enormous variety of plant species. Oak species dominate the highlands, while pistachio and almond are common at lower elevations. Also, hackberry, walnut, and pear trees are scattered throughout this eco-region (The United Nations Educational, Scientific and Cultural Organization (UNESCO), 2015). Due to the bees feeding from different and diverse plant species in Dena Biosphere Reserve that are mainly medicinal plants like Echium amoenum, Thyme, Almond, Eryngium, Astragalus, Mentha pulegium, Yarrow (Achillea millefolium), Common sage (Salvia officinalis), Coriander, Mint, Peppermint, Chicory, and Prangos ferulacea, the taste of the honey is unique and it has high medicinal properties and might cures various diseases (The sound of the extinction of Dena medicinal plants, 2017).

The current study aimed at finding the effect of local application of honey produced in Dena Biosphere Reserve on post-operative wound healing in the patients with pilonidal cyst excision by comparing two groups of patients, the first group, with honey gauze dressings, and the other group, with placebo gauze dressings. Comparison of these two groups would show whether such dressings can facilitate a faster wound healing, return to daily activities, changing the pain intensity, odor, discharge at the site of surgery, use of analgesics, and occurring of any side effects including infection, erythema, and bleeding.

## Materials and methods

### Design and setting

This study was designed as a non-commercial randomized placebo-controlled parallel assignment interventional (clinical trial) study in accordance with the principles of International Conference on Harmonization and Good Clinical Practice guidelines (ICH-GCP) (Dixon Jr., 1998). Blinding did not exist at the level of the investigator or the surgeon who will record the data post-operatively. Blinding was done at the patients’ level. The study was conducted at the surgical ward of Shahid Beheshti Hospital affiliated to the Yasuj University of Medical sciences, Yasuj, Iran, and was consisted of the 48 patients who underwent surgical resection for PSD with secondary intention healing. The project was found to be in accordance to the ethical principles and the national norms and standards for conducting research in Iran with the approval ID and date of IR.YUMS.REC.1399.088 and 2020.05.30 respectively.

### Main surgical outcomes

The time to complete wound healing, pain intensity, odor, discharge at the site of surgery, use of analgesics and the time of to return to the daily activities, and occurring of any side effects including infection, erythema, and bleeding were all recorded.

### Eligibility criteria

#### Inclusion criteria

**Fig. 2:**
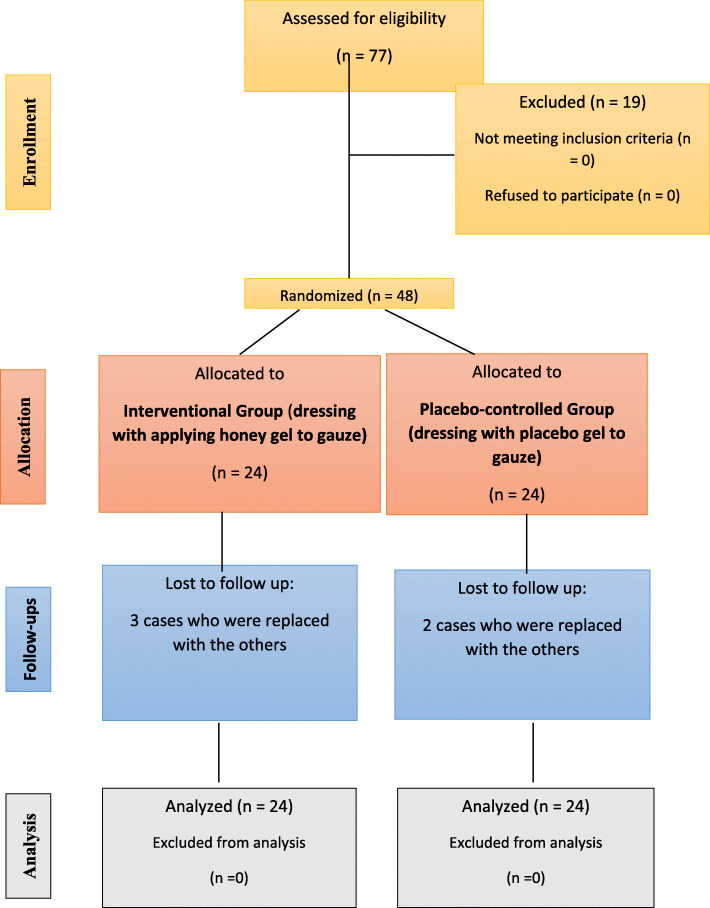
The CONSORT flow diagram

There were 77 patients who were assessed for eligibility criteria, of whom 48 cases complete the eligibility criteria. Inclusion criteria were as surgical excision of PSD for the first time, the patients with age 16 to 45 years old, body mass index between 20 and 30 kg/m^2^, and reading, completing, and assigning the consent form for participating in the study. The Consolidated Standards of Reporting Trials (CONSORT) flow diagram is shown in Fig. 2.

#### Exclusion criteria

Pregnant and lactating women, obese patients with body mass index > 30 kg/m^2^, addiction to drugs and alcohol, abnormal bleeding from the wound, using drugs that affect wound healing such as corticosteroids, using antibiotics prior to the surgical resection, not continuing the proposed treatment until the end of the study or request to leave the study, severe allergy or anaphylactic shock caused by the treatment, recurrent pilonidal sinus, pilonidal abscess, having chronic diseases such as diabetes, heart disease, kidney disease and diseases of the immune system, malignancy, organ transplantation, connective tissue disease and known allergies to honey, and living in the rural areas with difficult access to health care. Five patients who did not complete the follow-ups were removed and replaced by other patients.

### Sample size

**Fig. 3:**
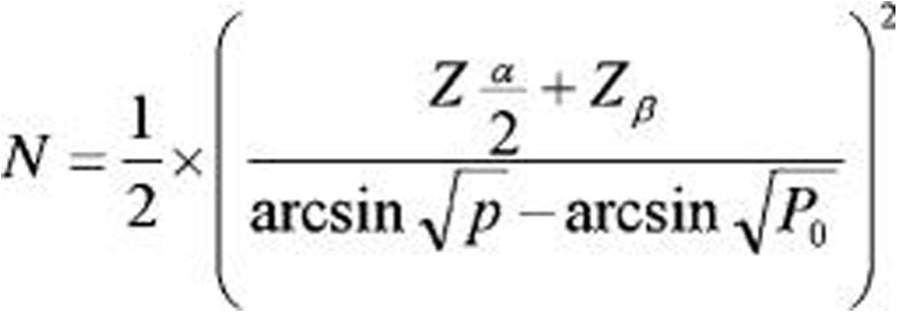
The formula used for sample size calculation

In a study done by Vasei et al., the percentage of wound infection in the control group was 100%, and in intervention group who had honey dressing was 0% (Vardi et al., 1998). According to mentioned study, the number of samples in each group was equal to 19 patients so that the efficiency of honey in 20% reduction of incidence of wound infection can be seen (95% confidence interval, and 80% power of study). Furthermore, the sample sizes were increased by 20% due to the possibility of dropping in the samples for various reasons. Finally, the total number of samples was 24 patients in each group. The formula used for sample size calculation was a bellow (Fig. 3) (Zhong, 2009).

## Methods

Both the consent and the questionnaire form were completed by the surgeon before the operation. One-gram cefazolin was injected intravenously half an hour before the operation in all the patients. All the patients underwent surgery with a similar technique (excision and healing by secondary intention). To add more, in our hospital (Shahid Beheshti Hospital affiliated to Yasuj University of Medical Sciences), the most common procedure and the usual method for the treatment of the PSD is the surgical removal with secondary intention wound healing. The surgical procedure was done under the spinal anesthesia. Patients were positioned in the prone jackknife position, and the buttocks were drawn to the side by an adhesive tape. Methylene blue was injected to define the exact borders of the pilonidal sinus, if required. An elliptical incision of the skin was made around the pilonidal sinus. For complete excision of the sinus or cyst, surgical diathermy was used up to the level of the fascia. The wound was covered with sterile gauze after hemostasis and irrigation with normal saline.

After the operation, at the surgery ward, at the time of discharge from the hospital, the patients were categorized in two groups (intervention and placebo-controlled groups) with no regard to the will of the researchers or the patients' condition and preference through drawing numbered sealed envelopes (random allocation). In both groups, wound was irrigated and washed with enough normal saline, and then in group one (intervention group), dressing with applying honey gel to gauze was done and in group two (placebo-controlled group), dressing with applying placebo gel to gauze was done. A pharmacist prepares both honey and placebo gels. The main element of honey medicinal gel is the unheated natural honey of Dena Biosphere Reserve in Kohgiluyeh and Boyer-Ahmad province located in the south-west of Iran.

The standards of the chemical and microbiological quality of the honey include maximum 5% sucrose, maximum 20% moisture, fructose to glucose ratio of at least 0.9, minimum pH of 3.5, culture for sulfite-reducing anaerobes (Clostridia) should be negative which shows no contamination, and culture for yeast and mold should be maximum 100 pieces in 1 g (Iranian National Standardization Organization, 2013). As a result, five different samples of natural unheated honey in this area were evaluated and amount of carbohydrates such as sucrose, fructose and glucose, and fructose to glucose ratio, moisture content, pH, and culture were assessed. Based on these findings and comparing them to the standards of the chemical and microbiological quality of the honey, the best type of honey was selected (Nikpour et al., 2014).

Gel is a topically used semi-solid structure with a good spreading and composed of small inorganic elements or large organic molecules penetrated by a fluid. Advantages of the gel are as simple manufacturing, giving a sense of cold, washing away easily after application, and providing protection with a formed thin layer. Gel is preferred over the cream, because it has a higher water content and can decrease the pain at the time of application, particularly when it is applied to the mucous membranes, and injured or burned tissue (Febriyenti et al., 2014).

A combination of carbopol (0.55 g), glycerin (5 g), methyl paraben (0.18 g), propyl paraben (0.02 g), triethanolamine (0.5 g), and distilled water (69.3 g) were used by the pharmacist to prepare the placebo gel (Goharshenasan et al., 2016). As a matter of fact, at first, carbopol is added to deionized and autoclaved water and kept in laminar flow condition for 24 hours. This product was then mixed (400 rpm/min) and other elements (glycerin, methyl paraben, propyl paraben, and triethanolamine were added. Afterward, invert sugar quantity was measured for standardization and adjustment (Nikpour et al., 2014).

According to Basson et al. study, a 25% honey concentration was effective on the most organisms, consequently, a 25% honey gel was produced in this study (Basson & Grobler, 2008). The same amount of elements, used for the preparation of the placebo, was used with the same quality in addition to 25 g honey for the preparation of the honey gel as a product.

**Fig. 4:**
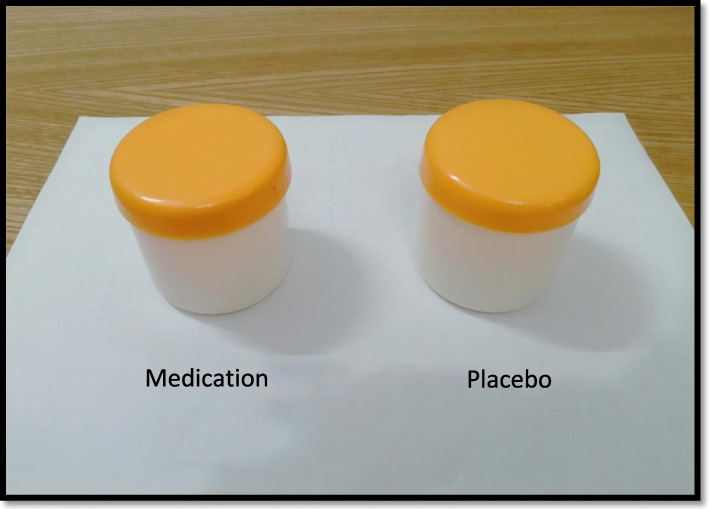
Medication and the placebo are provided to the surgeon as well as the patients with the similar appearance

At last, the final drug and placebo products were cultured and then used in the study if there were negative results (Nikpour et al., 2014). The produced gel and placebo were packed in wide-mouth high-quality plastic bottle containers with screw capped lid. Medication and the placebo are provided to the surgeon as well as the patients with the similar appearance (Fig. 4). Although honey is a unique good scent, but actually it was not obvious whether the patient is receiving honey or placebo through the smelling, because honey gel was a mixture of placebo ingredient and honey, which cause the honey gel not having its natural scent. To add more, blinding did not exist at the level of the investigator, but at the patients’ level.

In both groups, oral antibiotics were not prescribed after the discharge from the hospital. The patients were trained face to face for how to change the dressing at home (as the same way which was done at the time of discharge at hospital), wound care, personal hygiene, and nutrition before living the hospital. Patients' wounds were visited by the research resident and special nurse in prone position on the days 7, 15, 30, 45, 60, and 90 after surgery, and main outcome measures were evaluated by interview and clinical examination using a standardized form at the surgery ward. Moreover, the patients were asked to contact the research resident at any time during follow-up if they have concern about their wound. Healing time was defined as complete closure and epitheliazation of the wound and without any evidence of discharge, swelling, and cellulitis. At the first visit, in the first week after the operation, culture samples were taken from the wound secretion of all the patients and were sent to the laboratory of Shahid Beheshti Hospital for examination. If the culture was positive in the intervention or placebo-controlled group, the next culture was taken in the next visit. The duration of treatment in each group was based on the wound healing and was determined by the surgeon during the post-operative visits. Time-to-heal was provided as a mean or median time. The quantitative method of wound healing measurement was based on the reduction of wound volume, which was measured by the surgeon by pouring normal saline into the wound with the help of a 10–60 cc syringe on the follow-up visits on the days 7, 15, 30, 45, 60, and 90 and a decreasing trend in wound volume would indicate wound healing.

**Fig. 5:**
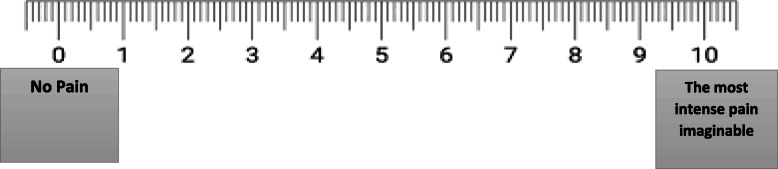
Visual analogue scale

The pain intensity of all individuals was measured through the visual analogue scale (VAS), which has good validity and reliability, on the days 7, 15, 30, 45, 60, and 90, respectively. The visual scale for measuring pain intensity is a 10-cm ruler with the word “no pain” written on the left end and “the most intense pain imaginable” on the right end (Fig. 5). The patient marks only one point on the continuum of the ruler, according to the amount of pain in the last 48 h. The amount of pain is measured by the surgeon using this scale as a standardized way for measuring pain. The linear-visual scale of pain is divided from 0 to 10 as follows: 0–1: no pain, 2–3: low pain, 4–5: high pain, 6–7: very bad pain, 8–9: maximum pain, 10 the most intense pain imaginable (Lazaridou et al., 2018; Carlsson, 1983). The amount of oral analgesics used in both groups is evaluated based on the number of analgesic pills used per day on the follow-up visits on days 7, 15, 30, 45, 60, and 90.

### Research bias

The best way of eliminating selection bias is to randomize the patients properly into the groups. In our study, randomization was achieved by categorizing the patients in two groups (intervention and placebo-controlled groups) with no regard to the will of the researchers or the patients' condition and preference through drawing numbered sealed envelopes (random allocation), and this gives every participant an equal chance to be allocated into any of the study groups. To avoid detection bias, we attempted to do the single-blind outcome assessment. In order not to have performance bias, the same care was provided in two groups (Fig. 4). Preventing information bias, standard measurement instruments and scales, e.g., questionnaires, visual analogue scale, and wound volumetry with measuring devices (syringe) by water.

### Ethical considerations

(1) One of the inclusion criteria of this study was completing the consent form to participate in the study; (2) If any side effects occurred due to the medication and placebo application, the administrator was responsible for that; (3) The use of medication and placebo should not inhibit or interfere with the patient’s main treatment, and is complementary; (4) The patient should not give any extra fee; (5) Permission from the University Ethics Committee was obtained.

### Statistical analysis

Statistical analysis was done using SPSS software Windows Ver. 21.0 (Chicago, IL, USA). Mean and standard deviation (SD) were used for quantitative variables, and percentage and frequency were used for qualitative variables to describe the data. A value of *P* less than 0.05 is considered statistically significant.

Data normality was examined by the Kolmogorov-Smirnov test. Comparison of quantitative data in the two groups was done using *t* test if there was normal distribution and Mann-Whitney and chi-square test were used otherwise. Qualitative data was reported using the chi-square test according to the odds ratio.

## Results

There were 77 patients who assessed for eligibility criteria, of whom 48 cases complete the eligibility criteria. There were 35 men and 13 women. Mean age was considered to be 25.41 ± 7.96, ranging 14–42 years.

Wound healing time (restoration and closure of the skin defect) was 78.0 ± 19.26 days, ranging 38–112 days in placebo-controlled group and 61.70 ± 16.50 days, ranging 28–91 days in intervention group (*p* value ≤ 0.01). The median time for wound healing in intervention and placebo-controlled groups were 73 and 56 days respectively (Table 4).

**Table 4:** Wound healing time

Wound healing time (days)	Mean	Median	Minimum	Maximum
Intervention group	61.70 ± 16.50	56	28	91
Placebo-controlled group	78.0 ± 19.26	73	38	112
*P* value	*p* ≤ 0.01			

**Fig. 6:**
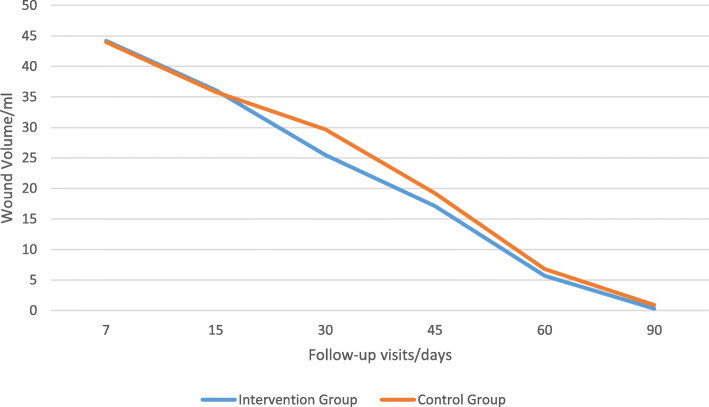
Mean wound volume in follow-up visits in two groups

The time to return to the daily activities was 28.62 ± 3.2 days, ranging 19–42 days in control group and 21.1 ± 2.4 days, ranging 13–33 days in intervention group (*p* value = 0.04). Mean wound volume in follow-up visits in two groups are summarized in Table 5 and Fig. 6.

**Table 5:** Mean wound volume in follow-up visits in two groups

Follow-up visits (days)/ml	7	15	30	45	60	90
Intervention group	44.2 ± 2.5	36.1 ± 2.8	25.5 ± 3.5	17.1 ± 1.8	5.7 ± 1.9	0.3 ± 0.4
Placebo-controlled group	44.0 ± 2.7	35.8 ± 2.8	29.7 ± 4.8	19.2 ± 1.6	6.8 ± 1.9	0.9 ± 0.8
*P* value	0.788	0.723	*p* ≤ 0.01	*p* ≤ 0.01	0.043	*p* ≤ 0.01

**Fig. 7:**
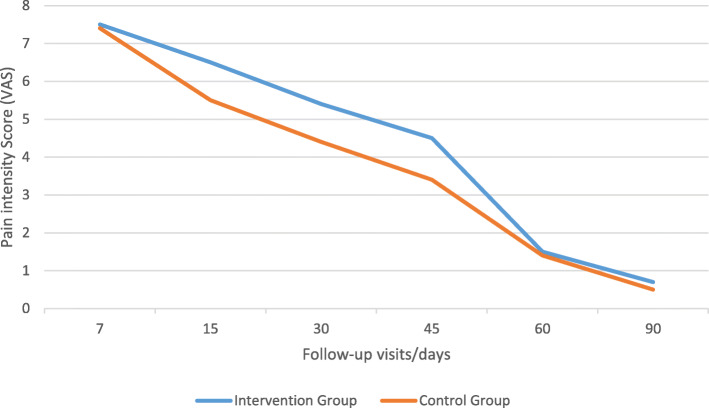
Mean post-operative pain level or intensity score according to VAS in follow-up visits in two groups

Post-operative pain level or intensity score according to VAS in follow-up visits in two groups are summarized in Table 6 and Fig. 7.

**Table 6:** Mean post-operative pain level or intensity score according to VAS in follow-up visits in two groups

Follow-up visits (days)/VAS for pain level	7	15	30	45	60	90
Intervention group	7.5 ± 0.5	6.5 ± 0.5	5.4 ± 0.5	4.5 ± 0.5	1.5 ± 0.5	0.7 ± 0.4
Placebo-controlled group	7.4 ± 0.5	5.5 ± 0.5	4.4 ± 0.5	3.4 ± 0.5	1.4 ± 0.5	0.5 ± 0.5
*P* value	0.573	*p* ≤ 0.01	*p* ≤ 0.01	*p* ≤ 0.01	0.258	0.146

**Fig. 8:**
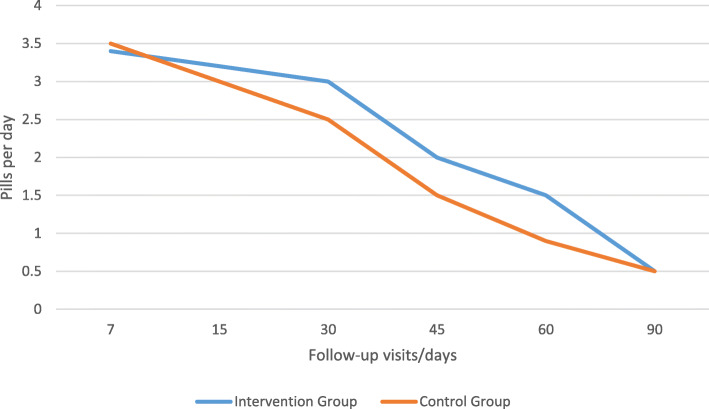
Post-operative analgesics consumption in follow-up visits in two groups

Post-operative analgesics consumption or the mean number of analgesic pills consumed per day after surgery for each group in the follow-up visit was summarized in Table 7 and Fig. 8.

**Table 7:** Post-operative analgesics consumption in follow-up visits in two groups

Follow-up visits (days)/pills per day	7	15	30	45	60	90
Intervention group	3.4 ± 0.5	3.2 ± 0.0	3.0 ± 0.0	2.0 ± 0.0	1.5 ± 0.5	0.5 ± 0.5
Placebo-controlled group	3.5 ± 0.5	3.0 ± 0.0	2.5 ± 0.5	1.5 ± 0.5	0.9 ± 0.2	0.5 ± 0.5
*P* value	0.397	*p* ≤ 0.01	*p* ≤ 0.01	*p* ≤ 0.01	*p* ≤ 0.01	1.000

**Fig. 9:**
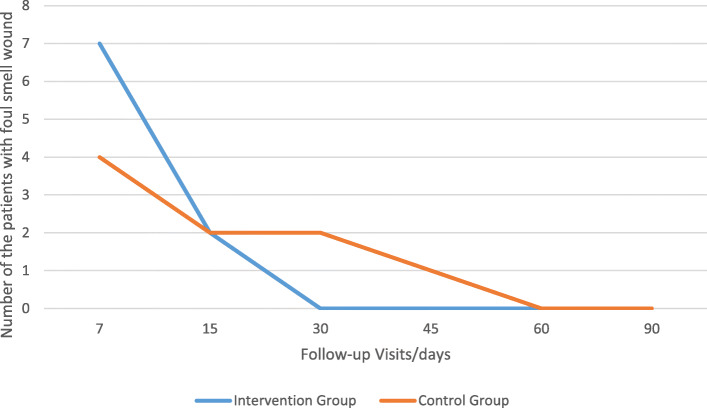
Frequency of the wound foul smell in the follow-up visits in two groups

Frequency of the wound foul smell was checked during the follow-up visits in the two groups and this was summarized in Table 8 and Fig. 9.

**Table 8:** Frequency of the wound foul smell in the follow-up visits in two groups

Follow-up visits (days)/number and percentage of the patients	7	15	30	45	60	90
Intervention group	7 (29.1%)	2 (8.3%)	0 (0.0%)	0 (0.0%)	0 (0.0%)	0 (0.0%)
Placebo-controlled group	4 (20.8%)	2 (8.3%)	2 (8.3%)	1 (4.1%)	0 (0.0%)	0 (0.0%)
*P* value	0.313	1.000	0.155	0.323	–	–

**Fig. 10:**
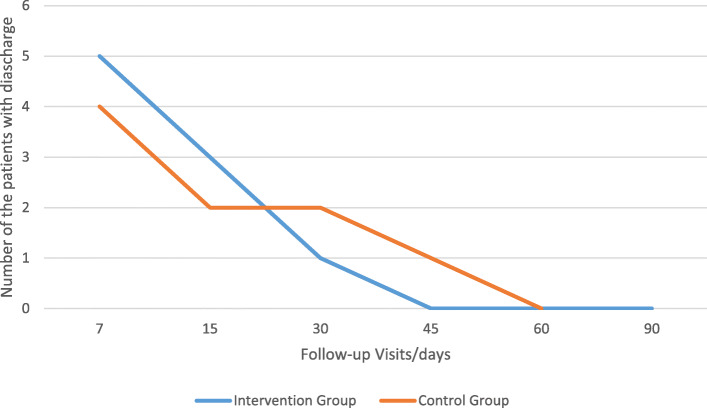
Fluid discharge at the site of surgery during the follow-up visits in the two groups

Fluid discharge at the site of surgery was checked during the follow-up visits in the two groups and this was summarized in Table 9 and Fig. 10.

**Table 9:** Fluid discharge at the site of surgery during the follow-up visits in the two groups

Follow-up visits (days)/number and percentage of the patients	7	15	30	45	60	90
Intervention group	5 (29.1%)	3 (8.3%)	1 (4.1%)	0 (0.0%)	0 (0.0%)	0 (0.0%)
Placebo-controlled group	4 (20.8%)	2 (8.3%)	2 (8.3%)	1 (4.1%)	0 (0.0%)	0 (0.0%)
*P* value	0.719	0.645	0.561	0.323	–	–

There was no occurring of any side effects and complications including infection, erythema, and bleeding in this study and culture was negative for all the patients at the first follow-up visit (7th day).

## Discussion

In the current study, there were 35 men and 13 women with the mean age of about 25 years. Our results were compatible with the results of other studies. The mean age at the presentation of the disease is 21 years in men and 19 years in women. The prevalence among men is two to four times that of women (Mahmood et al., 2020; Vasei & Jahangiri, 2008; Harris et al., 2012b).

In the placebo-controlled group, the duration of wound healing was 78 days [38–112] and in the intervention group, the duration of wound healing was 61.7 days [28–91]. A comparison of the duration of wound healing in the placebo-controlled group of this study and the duration of wound healing by the secondary intention obtained from other studies is summarized in Table 10 (Kronborg et al., 1985; Al-Hassan et al., 1990; Khawaja et al., 1992; Søndenaa et al., 1996; Hameed, 2001; Gencosmanoglu & Inceoglu, 2005).

**Table 10:** Comparison of the duration of wound healing in the placebo-controlled group of this study and the duration of wound healing by the secondary intention obtained from other studies

Study first author	Year of the study	Duration of wound healing by the secondary intention/days
Kronborg et al. (Kronborg et al., 1985)	1985	64 (17–157)
Al-Hassan et al. (Al-Hassan et al., 1990)	1990	91 (28–546)
Khawaja et al. (Khawaja et al., 1992)	1992	41 (not reported)
Søndenaa et al. (Søndenaa et al., 1996)	1996	70 (28–266)
Hameed et al. (Hameed, 2001)	2001	70 (59–91)
Gencosmanoglu et al. (Gencosmanoglu & Inceoglu, 2005)	2005	79 (21–112)
Salehi et al (our study)	2021	78 (38–112)

There was significant difference between intervention and placebo-controlled group according to the wound healing time and the time to return to the daily activities, and this shows that application of honey is effective in decreasing the time for healing after PSD excision and coming back to daily activities. Our results were compatible with the results of Vasei et al. study, in which new granulation tissue was seen at the operation site after two week from the surgery, but it was not compatible with that according to the wound healing time. In Vasei et al study, there was no significant association between intervention and placebo-controlled group according to wound healing time (Vasei & Jahangiri, 2008). In a study done by Al-Waili et al., 50 patients having post-operative wound infections following caesarean sections or total abdominal hysterectomies were evaluated in two groups using honey, and ethanol and povidone-iodine dressing respectively. They concluded that topical application of crude undiluted honey could help in faster eradication of bacterial infections, reduce period of antibiotic use and hospital stay, accelerate wound healing, prevent wound dehiscence and need for re-suturing and result in minimal scar formation (Al-Waili & Saloom, 1999). Misirlioglu et al. found that the donor sites dressed with honey are epithelized approximately 4.1 days faster than the donor sites dressed with saline-soaked gauzes (9.1 vs.13.2 days, statistically significant) (Misirlioglu et al., 2003). Dunford et al. applied honey daily directly on to the wound and found that the granulation and epithelial tissues were visible within one-week, significant healing took place within three weeks, and the wound healed completely after six weeks (Dunford et al., 2000). In a study done by Nikpour et al. using honey gel and placebo on the cesarean wounds, she found no significant change in the approximation of the wound on the 7th day of follow-up (*p* value = 0.311) and this was consistent with the results of this study (Nikpour et al., 2014).

According to the wound healing time, as we had a skewed distribution with extreme values (28–91 and 38–112 for the intervention and placebo-controlled groups respectively), and as the effect on the median is smaller, and the median is a better measure of central tendency than the mean, we used this measure, and the results showed a parallel result to the mean.

Mean wound volume was not significantly different at the 7 and 15 days of follow-up visits, but it was significantly lower in the intervention group at all of the next follow-ups, and this shows that the honey would affect the wound healing with almost a two weeks delay.

Mean post-operative pain level or intensity score was significantly higher in intervention group according to VAS in all follow-up visits except the day 7, and this shows that application of honey is related to cause more pain and discomfort, and this result is compatible with the result on post-operative analgesics consumption in which significantly more usage of analgesics were seen in intervention group at the 15, 30, 45, and 60 follow-up days. There is controversy about pain and honey dressings, as there are some reports on the pain reliving or inducing effects of using honey (Søndenaa et al., 1996). Our results were compatible with the results of Vasei et al report in which pain was much exacerbated with the use of honey dressing and last for 2 h, but pain was not increased in the placebo-controlled group with the use of alcohol and betadine solution (Vasei & Jahangiri, 2008). Misirlioglu et al. found less pain with honey-soaked gauzes than with paraffin gauzes and this was comparable with our study (Misirlioglu et al., 2003). Dunford et al. described the pain in a patient with leg wound when the honey was first applied, which eased within 20 or 30 min as a ‘drawing’ sensation in the wound (Dunford et al., 2000). It has been proposed that high osmolarity of honey was not the reason for the pain, because in the patients who have found honey painful, the sugar solutions with the similar osmolarity to honey have not caused pain. Another finding is that the certain wounds are sensitive to the honey’s acidity (low pH). Pain was not found in the patients who have tried using pH neutralized honey (Dunford et al., 2000). So, we could say that the reason for the pain could be the honey’s acidity, and the organic chemicals which stimulates the sensitized and hyper-responsive nerve endings (nociceptors) (Molan & Betts, 2004).

Although application of honey decreased frequency of the wound foul smell in the intervention group from seven to two cases at the day 15 follow-up, or fluid discharge in the intervention group at the site of surgery from five cases to one case at the day 30 follow-up, there was no significant difference between intervention and placebo-controlled group according to the foul smell and fluid discharge at the site of the operation. By the way, culture was negative for all the patients at the first follow-up visit (7th day). Our results were comparable with Vasei et al study, in which there was no patient with foul smell fluid discharge at the site of the operation in intervention group, but foul smell wound with whitish discharge in 3 patients in placebo-controlled group (Vasei & Jahangiri, 2008). Vardi et al. showed that honey is useful in the treatment of post-surgical wounds that are infected and do not respond to conventional systemic and local antibiotic treatment (Vardi et al., 1998). Grant found that the dressing with honey, could manage the wound exudate levels, and that the wound odor appeared to be well controlled in all the cases, and she concluded that the use of honey dressing on the post-surgical pilonidal sinus wounds could potentially improve the clinical outcomes, reduced treatment time, and a faster return to the former quality of life (Grant, 2009). The strong osmotic action of the honey is the reason for which it could (1) minimize the unpleasant smelling of the wounds, (2) draw out exudates and lymph fluid from the wound towards the surface, (3 add the moisture needed for autolytic debridement, and (4) provide a large quantity of glucose as a substrate rather than amino acids for the bacterial metabolism. It means that in the absence of the honey, bacteria would change amino acids to the malodor ammonia, amines, and sulfur compounds which are responsible for the malodorous wound, but in the presence of the honey, bacteria would use glucose instead and change it to the odorless lactic acid (Alam et al., 2014).

There were no side effects and complications in both groups of the current study, and this was comparable with the study done by Vasei et al, in which 7 patients out of 12 cases in intervention group developed with bleeding 2 h after the operation with the using of honey dressing (Vasei & Jahangiri, 2008). No toxic effects have been reported in the literature for using honey (Dunford et al., 2000).

## Study limitations

The usage of honey has some advantages as well as some disadvantages. The advantages of using honey have been previously discussed in Table 1, and the disadvantages of using honey and the limitations of the study are gathered in Table 11.

**Table 11:** Disadvantages and limitations of using honey as a dressing in our study

Disadvantages of using honey	Limitations of the study
High price	The effects of various types of honey and their concentration were not studied yet
Becomes more fluid at high temperatures, and it may liquefy at ambient wound temperature, and risk of leakage	Some of the patients in intervention and placebo groups were lost to follow-up, and replaced with the other ones.
Risk of liquefaction restricts body site usage	The PSD tissue was not evaluated by the computed tomography (CT) scan or magnetic resonance imaging (MRI).
Due to bacterial inoculation of the wound from unsterilized honey, sterilization of unprocessed honey is needed	For the widespread use of honey, appropriate sterilization on unprocessed honey is needed (i.e., gamma-irradiated), and in this study we could not apply that
Could cause pain and discomfort	
Gamma-irradiated for sterilization of unprocessed honey is expensive	

## Recommendations

It has been suggested that some further studies are needed to make honey standardized for the treatment and to evaluate the dosage, duration and cost-effectiveness of the treatment. A randomized controlled trial is the gold standard to test efficacy in comparison with standard therapy and is proposed as the way forward in further studies on the use of honey in wound care.

## Conclusions

In the intervention group, there was significantly lower wound healing time, the lower time to return to the daily activities, lower mean wound volume at the days of 30, 45, 60, and 90 of the follow-up, higher mean post-operative pain level at the days of 15, 30, 45, 60, and 90 of the follow-up, and more usage of analgesics at the days of 15, 30, 45, and 60 of the follow-up. There was no significant difference between intervention and placebo-controlled group according to the foul smell and fluid discharge at the site of the operation. There were no side effects and complications in both groups of the study.

Pilonidal sinus disease represents a significant disease burden, disturbing mostly the young individuals with pain, discomfort, restricting the physical activity, and enormous socioeconomic consequences. The perfect treatment would be a fast cure allowing the patients to return rapidly to their normal activities, with least morbidity and complications. In addition to the complications like surgical wound infections, the care services are too expensive. These difficulties raised new attentions to the traditional remedies.

To conclude, application of honey after resection surgery with secondary wound healing is associated with a better surgical outcome and could eventually decrease healing time and reduce duration of return to normal activities, but could increase post-operation pain and analgesic consumption, and no effect on foul smell and discharge.

## Data Availability

All data generated or analyzed during this study are included in the manuscript.

## Abbreviations

CONSORT: Consolidated Standards of Reporting Trials
g: Gram
IL-6: Interleukin 6
ICH-GCP: International Conference on Harmonization and Good Clinical Practice guidelines
MM6: Mono-Mac 6
NO: Nitric oxide
PSD: Pilonidal sinus disease
PHMB: Polyhexamethylene biguanide
SD: Standard deviation
TNF-α: Tumor necrosis factor alpha
VAS: Visual analogue scale
CT scan: Computed tomography
MRI: Magnetic resonance imaging
